# Gear Shape Parameter Measurement Using a Model-Based Scanning Multi-Distance Measurement Approach

**DOI:** 10.3390/s20143910

**Published:** 2020-07-14

**Authors:** Marc Pillarz, Axel von Freyberg, Andreas Fischer

**Affiliations:** Bremen Institute for Metrology, Automation and Quality Science (BIMAQ), University of Bremen, 28359 Bremen, Germany; a.freyberg@bimaq.de (A.v.F.); andreas.fischer@bimaq.de (A.F.)

**Keywords:** optical gear measurements, model-based scanning multi-distance measurements, confocal-chromatic sensor, large gear, measurement uncertainty of gear parameters

## Abstract

To reduce wind turbine failures by defective drive trains, deviations in the geometry of large gears (diameter ≳ 1 m) must be extensively determined with single-digit micrometer uncertainties. Fixed measuring volumes limit standard measuring methods like coordinate and gear measuring instruments for large gear measurements. Therefore, a model-based scanning multi-distance measurement approach for gear shape parameters is presented. The measurement approach has a scalable design and consists of a confocal-chromatic sensor, rotary table as a scanning unit and model-based signal processing. A preliminary study on a midsize spur gear demonstrates the general feasibility of the model-based scanning multi-distance measurement approach. As a result, the mean base circle radius as the fundamental gear shape parameter is determined with an uncertainty of <5 μm. The calibration and adjustment of the sensor arrangement were performed with a known calibration gear. Scalability is not experimentally validated in this article. However, simulations verify the scalability of the measurement approach in a first step. For gears with 1 m in diameter and varying tooth flank geometries, the estimated achievable uncertainty of the mean base circle radius is still <5 μm. Therefore, the model-based scanning multi-distance measurement approach is a promising alternative for gear inspection.

## 1. Introduction

### 1.1. Motivation

Defects of the drive train are a significant cause of wind turbine failures [1]. The base circle radius of a spur gear correlates with the profile slope deviation and is the shaping parameter for an involute geometry. The involute geometry of the teeth of a spur gear is decisive for a uniform power transmission. Deviations of the base circle radius from the nominal geometry, which correlate to the profile slope deviation, lead to premature wear of the gears. To reduce drive train defects, reliable quality assessments of the gear shape parameters such as the base circle radius, profile, lead, pitch and runout deviations of large gears with diameters ≳ 1 m are required [2,3,4]. However, measurements of large gears represent a major challenge in dimensional metrology. According to ISO 1328-1 and the golden rule of metrology, a measurement uncertainty of ≈5 μm is required for gear quality grade 5 to quantify the base circle radius of a gear with a diameter of 890 mm and a module of 10 mm. The mass and the dimensions of large gears are associated with a high logistical effort when measuring large gears [5]. The measurement of large gears of different sizes requires measuring systems with scalable measuring volumes. Besides, in the production of large gears compared to small gears, the larger chip volume and longer machining time result in asymmetrical heat input and noticeable tool wear. Therefore, the standard gear tests with a random test scope of four teeth are not sufficient for large gear measurements [6]. It cannot be assumed that the individual teeth have nearly the same shape and surface quality. To evaluate the shape parameters of large gears, the geometry data of all teeth must be taken into account. This means that extensive measurements of all teeth over the whole circumference are required. For this reason, a scalable gear measurement system that acquires extensive data from all teeth is desired to quantify shape parameters such as the base circle radius of large gears with single-digit micrometer uncertainty.

### 1.2. State of the Art

At present, coordinate measuring machines (CMM) and gear measuring instruments, with mainly tactile probes, are standard measuring systems to quantify gear geometry [2,7,8]. However, due to an individually limited measuring volume, the standard measuring systems are only conditionally suitable for extensive quality assessments of the geometry of large gears.

While no significant developments are expected in the field of tactile gear metrology [9], optical sensors feature high resolutions up to a few nanometers and enable, fast measurements and flexible, scalable measurement set-ups. Indeed, optical sensor systems have been recently used and investigated for extensive gear measurements, although they have complex sources of error [3]. Therefore, the subsequently described state of the art is focused on optical sensor approaches for gear measurements and. The current state of the art is evaluated regarding the criteria of measurement uncertainty and scalability.

Optical measurement approaches for the non-contact detection of surface contours are often based on triangulation principles and can perform point-by-point, line-wise and area-oriented measurements. Younes et al. introduced in 2005 a point-by-point triangulation laser for profile geometry measurements of small gears. However, the required measurement uncertainty to assess the tolerances for gear quality grade 5 according to ISO 1328-1 was not achieved [10]. The measuring system is also not intended for scalable large gear measurements. Measurement concepts for gear measurements based on laser line triangulation sensors were presented in [11,12]. Auerswald et al. presented in 2019 a laser line triangulation approach for large helical gear measurements. Auerswald et al. attains measurement uncertainties of the profile slope deviation of 8.2 μm (*k* = 1). The uncertainty is not sufficient to quantify the desired tolerances of large gears. Which is not sufficient to quantify the desired tolerances of large gears. The scalability of the measuring system for large gear analysis is not discussed. In 2020, Guo et al. show the successful measurement of the total profile deviation of a small gear (*d* = 8 mm) with one laser line triangulation sensor in combination with a rotary table. The total measurement uncertainty amounts to 1 μm (a coverage factor was not specified). However, scalable measurements of large gears were not discussed. Nikon introduced the HN-C3030 3-D gear measuring system, which is based on fringe projection combined with computerized numerical control of the motion [13]. A maximum permissible error of MPE_E_ = (1.6 + 4 *L*/1000) μm (*L* = measured length in mm) is indicated. Due to a fixed measuring volume for gear diameters up to 300 mm, the HN-C3030 is not suitable for large gears. Peters et al. presented in 2000 a non-scalable fringe projector in combination with a rotary table to determine the tooth-individual surface geometry of helical gears [14]. They achieved uncertainties of slightly < 10 μm (a coverage factor is not specified). In 2006, Meeß et al. introduced an area-oriented approach for gear measurements using stripe pattern projection. They achieved measurement uncertainties of the profile slope deviation of *U* = 17.9 μm (*k* = 2) [15]. The measuring system presented by Meeß et al. is also not scalable. The use of the moiré technique in the form of projection moiré is another area-oriented approach which was recently investigated for gear measurements [3,16]. Sciammarella et al. demonstrated in 2005 that projection moiré achieves comparable results to coordinate measuring machines when measuring gear contours. The contours of the profile lines of a gear were measured with an uncertainty of < 2.6 μm (a coverage factor was not specified). Information on measurement uncertainties of gear parameters were not given. Chen et al. detected the 3-D topology of the tooth flanks of a spur gear and determined the involute profile deviation and lead profile deviation. The achieved measurement uncertainty for the profile deviation amounts on average to 2.67 μm (a coverage factor was not specified). The mean lead profile uncertainty was 2.81 μm (a coverage factor was not specified). The obtained measurement results showed great potential for gear measurements. However, an application for large gears and therefore a scalable approach was not mentioned. In summary, the current triangulation approaches are not validated for scalable large gear measurements.

Besides triangulation, interferometry is also used for optical gear measurement. In 2014, Fang et al. presented an interferometric measurement approach for capturing the geometry of gears [17]. Systematic deviations due to position deviations of the gears were compensated, so that results close to the reference were achieved. However, quantitative information about the achieved measurement uncertainty was not given. Furthermore, the system is not designed for large gears. Balzer et al. used a frequency-modulated interferometric sensor in combination with a CMM in 2015 [6]. The beam path is guided via fiber optics into a sensor head, and the sensor head is moved by the coordinate measuring machine. Standard measurement uncertainties of 0.5 μm (*k* = 1) were observed [2]. However, the measuring system can only be scaled to a limited extent within the measuring volume of the CMM. Hence, current interferometric sensor approaches are not suitable for large gear measurements.

Confocal-chromatic distance sensors are another promising optical sensor concept for gear measurement. Confocal-chromatic sensors are less surface-dependent, so that even the reflective and curved tooth flank surfaces can be detected. Moreover, distance uncertainties in the low single-digit micrometer range are achieved independently of the surface. Measuring rates of up to 70 kHz are possible, making dynamic measuring concepts feasible [18]. Reports on gear measurement capability are still pending.

In summary, the current state of the art of optical sensors shows the potential for optical gear measurement approaches in general. At present, however, there is no scalable optical approach for extensive large gear measurements that achieves the required uncertainty for the gear parameters.

### 1.3. Aim and Structure of the Article

In this article, a model-based scanning multi-distance measurement approach is presented to quantify the mean base circle radius as a fundamental shape parameter of large gears with an uncertainty below 5 μm (*k* = 1) (which corresponds to an uncertainty of 1 μm for the profile slope deviation). The measurement approach is realized by using a confocal-chromatic sensor in combination with a rotary table to perform continuous distance measurements to all tooth flanks. A flexible sensor arrangement enables a scalable measuring volume. Note that in this article the scalability is not investigated experimentally. This will be subject of future research. The shape parameter mean base circle radius is evaluated as model-based according to the geometric data of all teeth. In addition to the initially considered base circle radius, further deviation parameters like the standardized profile slope deviation can be evaluated, which is planned in future work.

The measurement principle of the model-based scanning multi-distance measurement approach is introduced in Section 2. The experimental set-up is then described in Section 3, and the experimental results on a midsize spur gear with an uncertainty < 5 μm (*k* = 1) follow in Section 4. As a result, the model-based scanning multi-distance measurement approach for gear inspection is validated. The scalability of the measuring concept for large gears is then initially verified by simulations in Section 5. The article closes with a conclusion and an outlook in Section 6.

## 2. Measurement Principle

### 2.1. Measurand Base Cricle Radius

In this article, the mean base circle radius *r*_b_ over all teeth of midsize spur gears is measured to quantify the deviations of the involute geometry. The base circle radius is the shaping parameter for the geometry of the tooth flanks. Usually the base circle radius is not directly evaluated in standard gear measurements. Instead, the profile slope deviation is used to evaluate the tooth flank geometry. However, the base circle radius correlates with the profile slope deviation. The actual mean base circle radius of a non-modified spur gear

(1) $$r_{b}=r_{b,n}\cdot \frac{{\bar{f}}_{H\alpha }}{L_{\alpha }}+r_{b,n}$$

can be calculated as a function of the nominal base circle radius *r*_b,n_, the mean profile slope deviation ${\bar{f}}_{H\alpha }$ and the evaluation range *L*_α_. The profile slope deviation defines the deviation of the actual slope compared to the nominal slope [19]. To validate the model-based scanning multi-distance measurement approach, a reference value for the base circle radius is thus obtained from the profile slope deviations detected with tactile reference measurements and by applying Equation (1).

### 2.2. Model-Based Evaluation of the Base Circle Radius

**Figure 1:**
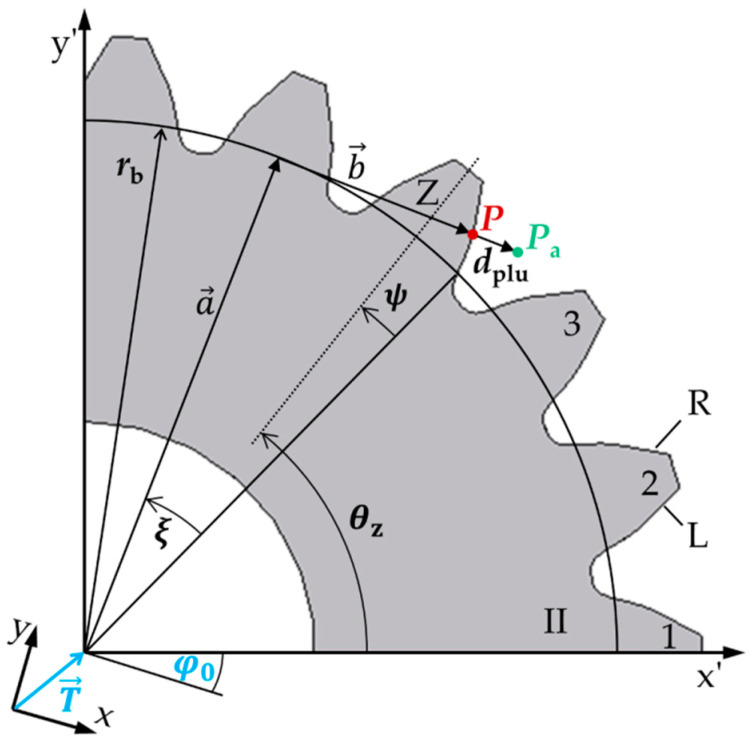
Geometric model for determining the shape parameter base circle radius *r*_b_ of a non-modified spur gear with an involute profile, based on a measuring point *P*_a_, the root point *P* of the nominal gear geometry for the plumb line distance *d*_plu_ and the position parameters $\xi ,\theta_{z},\psi_{b},\vec{T},\varphi_{0}$. Note, here, the plumb line distance of the measuring point to the nominal geometry of the gear is illustrated enlarged.

To determine the base circle radius *r*_b_ of a spur gear, an evaluation method based on the actual profile geometry of the tooth flank is presented. Figure 1 shows the geometry of a gear in the transverse section in a measuring coordinate system (*x, y*) that is translated and rotated relative to the workpiece coordinate system (*x’, y’*).

According to [9,20,21], a measured actual point

(2) $$P_{a}=\left[\begin{array}{c} x_{a}\\ y_{a} \end{array}\right]=P+\frac{d_{\mathrm{plu}}}{\left|\vec{n}\right|}\cdot \vec{n}$$

on the individual tooth flank can be described by adding the nominal point *P* of a spur gear to the deviation to the nominal geometry in the normal direction $\vec{n}$ of the surface, the plumb line distance *d*_plu_. According to Figure 1, each nominal point

(3) $$P=\left[\begin{array}{c} x_{p}\\ y_{p} \end{array}\right]=\vec{a}+\vec{b}+\vec{T}=r_{b}\cdot \left[\begin{array}{c} \cos\left(\xi +\theta_{z}-\psi_{b}+\varphi_{0}\right)\\ \sin\left(\xi +\theta_{z}-\psi_{b}+\varphi_{0}\right) \end{array}\right]+r_{b}\cdot \xi \cdot \left[\begin{array}{c} \sin\left(\xi +\theta_{z}-\psi_{b}+\varphi_{0}\right)\\ -\cos\left(\xi +\theta_{z}-\psi_{b}+\varphi_{0}\right) \end{array}\right]+\vec{T}$$

of tooth *Z* of a spur gear can be calculated by vector addition of a radial component $\vec{a}$, a tangential component $\vec{b}\;$ in the workpiece coordinate system (*x’, y’*) and the translation vector $\vec{T}={\left[x_{t},y_{t}\right]}^{T}$ to the workpiece coordinate system. The length of the radial vector $\vec{a}$ is equal to the base circle radius *r*_b_ of the gear and the angle of $\vec{a}$ is $\delta =\xi +\theta_{z}-\psi_{b}+\varphi_{0}$ in the measuring coordinate system. The tangential vector $\vec{b}$ has a length of $r_{b}\cdot \xi$ with an appropriate angle of $\delta -\frac{\pi }{2}$. The parameter ξ defines the rolling angle of the gear assigned to the nominal point *P*. The center axis of a tooth Z is described by the angle *θ*_z_. The angle $\psi_{b}$ is the base tooth thickness half angle. According to Equations (2) and (3), a measured actual point on the involute thus depends on the geometry parameter $r_{b}$, the position parameters $\xi ,\theta_{z},\psi_{b},\vec{T},\varphi_{0}$ and the plumb line distances *d*_plu_. Hence, to determine the base circle radius of a gear, the inverse problem

(4) $$r_{b}=f\left(P_{a},\xi ,\theta_{z},\psi_{b},\vec{T},\varphi_{0},d_{\mathrm{plu}},\vec{n}\right)$$

must be solved. Note, however, that the parameters $r_{b},\;\xi ,\vec{T},\varphi_{0}$ are unknown. The inverse problem is therefore under-determined. To solve the inverse problem, at least four measuring points are required according to the existing unknown parameters to get a determined system of equations.

An iterative approach is used for the determination of the actual base circle radius by approximating an ideal involute into the measured points. By minimizing the sum of the squared plumb line distances

(5) $$\begin{array}{c} \min\\ r_{b},\;x_{t},\;y_{t},\;\varphi_{0} \end{array}\left(\overset{n}{\underset{i=1}{\sum }}d_{\mathrm{plu},i}^{2}\right)$$

to the measured points according to the least-squares method, the ideal involute is calculated depending on the parameters $r_{b},\;x_{t},\;y_{t},\;\varphi_{0}$.

According to [20,22], the plumb line distances to the ideal involute can be calculated to

(6) $$d_{\mathrm{plu},i}=r_{b}\cdot \left(\sqrt{\frac{r_{I}^{2}}{r_{b}^{2}}-1}-fladir\cdot \left(\gamma -\theta_{z}+\psi_{b}-\varphi_{0}+\arctan\left(fladir\cdot \sqrt{\frac{r_{I}^{2}}{r_{b}^{2}}-1}\right)\right)\right),$$

whereby the corresponding rolling angles $\xi_{i}$ to the root point of the plumb line distances on the involutes are determined implicitly. The parameter *r*_I_

(7) $$r_{I}=\sqrt{{\left(x_{I}-x_{t}\right)}^{2}+{\left(y_{I}-y_{t}\right)}^{2}}$$

is the radius of the detected actual point in polar coordinates within the workpiece coordinate system and

(8) $$\gamma =\arctan\left(\frac{y_{I}-y_{t}}{x_{I}-x_{t}}\right)$$

describes the corresponding polar angle to the radius *r*_I_. The term *fladir* represents a factor for the flank side (left side: −1, right side: 1). Hence, the actual base circle radius of the gear is determined by using Equation (5).

### 2.3. Model-Based Multi-Distance Measurement Approach

**Figure 2:**
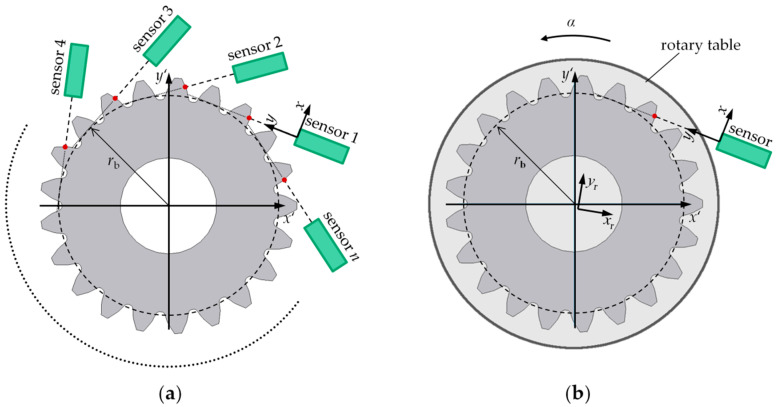
Model-based multi-distance measurement principles consisting of (**a**) a multisensory system with *n* optical distance sensors in a common measuring coordinate system (*x*, *y*) measuring the tooth contour of a non-modified spur gear with a workpiece coordinate system (*x’*, *y’*) and (**b**) a single-sensor system (*x*, *y*) in combination with a rotary table (*x*_r_, *y*_r_) measuring continuously the tooth contour of a spur gear (*x’*, *y’*) depending on the rotation angle *α*.

For the model-based evaluation of the base circle radius of a spur gear, the contour of the tooth flanks must be measured. For this purpose, two optical multi-distance measurement approaches are introduced in Figure 2.

Figure 2a introduces a multisensory approach for model-based multi-distance measurements of gear geometry. *n* ≥ 4 optical distance sensors (at least four sensors are necessary to obtain a determined system of equations) are used to perform a static gear measurement to characterize the gear geometry. The sensors are distributed around the circumference and aligned tangentially to the nominal base circle in the transverse section of the gear. The measuring volume can be scaled according to the size of the measured object by a flexible positioning of the sensors. However, the exact positions and alignments of the sensors are not known. Therefore, the sensors are adjusted in terms of the sensor positions and alignments in a common measuring coordinate system. Each sensor measures a vertical distance to the surface of a tooth flank. The measured distances are then converted into coordinates in the measuring coordinate system. A mean base circle radius over the measured teeth can be determined according to the model-based evaluation introduced in Section 2.2.

The multisensory approach is particularly suitable for a scalable and mobile measurement approach for gear measurement. The use of several sensors allows parallel data acquisition, which results in short measurement times. With previous knowledge, the mean base circle radius can be calculated. However, only the mean shape parameters of the gear can be determined. Statements about the individual geometry of teeth are not possible or only to a limited extent and are associated with a high number of sensors (number of teeth times four). The use of many sensors is, however, not economical and involves a great deal of effort in implementation and arrangement. The uniformity of several sensors must also be considered.

A model-based multi-distance measurement approach for gear measurements consisting of a single optical distance sensor in combination with a rotary table is depicted in Figure 2b. The rotary table and the sensor form a common measuring coordinate system in the center of rotation. Here, the sensor system is calibrated and adjusted with a known gear, since the exact sensor position and alignment are not known. An unknown sensor arrangement leads to a systematic error when calculating the base circle radius. The gear to be measured is positioned as concentrically as possible on the rotary table. The sensor is also aligned tangentially to the nominal base circle in the transverse section of the gear. In comparison to the multisensory approach, a dynamic gear measurement is performed. The contours of the tooth flanks are continuously measured in the form of distances *d*_i_ depending on the rotation angles *α*_i_. The scanning data acquisition results in an over-determined system of equations, which enables the iterative evaluation of the shape parameter mean base circle radius according to Equation (5).

The scanning data acquisition over the whole circumference enables also the evaluation of tooth individual shape parameters, like the individual base circle radius, the profile slope deviation and so on. Besides, the evaluated shape parameter can be averaged over more measuring points, which reduces random errors. Due to the use of a rotary table, further uncertainty contributions like a wobble or eccentricity must be considered due to the positioning of the gear. Also, the sensor arrangement of the alternative measurement approach is flexible, allowing a scalable measurement set-up. Due to the necessity of a rotary table, however, the flexibility is slightly limited. In this article, the single-sensor approach is applied to validate the model-based scanning multi-distance measurement approach for the measurement of gear shape parameters.

## 3. Experimental Set-up

The feasibility study of the model-based scanning multi-distance measurement approach is shown under economically efficient boundary conditions using the example of the single-sensor approach, since a confocal-chromatic distance sensor and rotary table are available. In addition, the scanning data acquisition of the entire tooth flank contours allows more measuring points to be recorded. This aspect offers more degrees of freedom when evaluating the shape parameter mean base circle radius. The experimental set-up is described in the following section.

### 3.1. Measurement Objects

**Figure 3:**
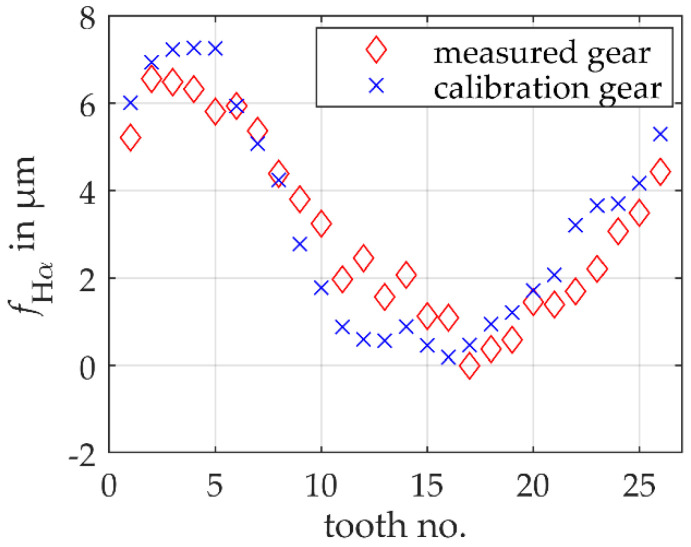
Profile slope deviations $f_{H\alpha }$ of the individual teeth of the gear to be measured and the calibration gear measured with a coordinate measuring machine.

For a general validation of the model-based scanning multi-distance measurement approach for gear measurements, a midsized non-modified spur gear is used. The gear has 26 teeth with an involute profile, a normal module of 3.75 mm and a nominal base circle radius of 45.8100 mm. To validate the model-based scanning multi-distance measurement approach, a reference measurement of the gear is performed with a Leitz PMM-F 30.20.7 portal coordinate machine. With a standard gear measurement, the profile slope deviations of all teeth are measured with an uncertainty of 1 μm for *k* = 1. Based on Equation (1), a reference mean base circle radius is calculated to 45.8182 mm ± 0.0005 mm (*k* = 1). Note that the small uncertainty results from averaging over all teeth. The tooth individual shape deviations are shown in Figure 3 using the example of a profile slope deviation $f_{H\alpha }$. The profile slope deviation is nearly sinusoidally distributed over the circumference of the gear. The sinusoidal distribution results mainly from a systematic error in the measurement of the workpiece coordinate system during the reference measurement of the gear, which is superimposed on the production-related shape deviations.

A known gear is used to calibrate the model-based single-sensor multi-distance measurement approach. According to ISO 15530, the calibration gear must fulfill the similarity condition concerning the gear to be measured. For this reason, a midsized non-modified spur gear with nominally the same geometry with 26 teeth with an involute profile, a normal module of 3.75 mm and a nominal base circle radius of 45.8100 mm is used. The reference mean base circle radius is 45.8184 mm ± 0.0005 mm (*k* = 1) and thus deviates on average by 0.2 μm from the gear to be measured. The tooth individual shape deviations are also shown in Figure 3. Here, too, an approximately sinusoidal distribution of the profile slope deviations over the entire circumference of the gear can be seen, which results from a superposition of individual shape deviations and a systematic error from the reference measurement. Accordingly, the same systematic influences are observed in both reference measurements. Even if the individual tooth shape deviations of the two gears differ quantitatively, they are almost identical on average (see reference mean base circle radii). The residuals of ideal and actual involutes also have similar shapes. The condition of similarity is therefore largely met concerning the actual geometry.

### 3.2. Measurement Object and Sensor Arrangement

**Figure 4:**
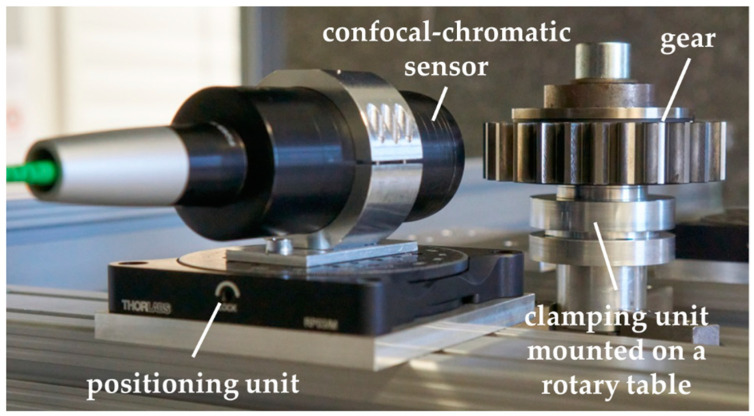
Experimental set-up for model-based scanning single-sensor multi-distance gear measurements of the mean base circle radius of a non-modified involute spur gear with a nominal base circle radius of 45.8100 mm. The single-sensor approach consists of a confocal-chromatic distance sensor and a rotary table to perform scanning measurements of the tooth flank contours.

The experimental set-up of the model-based scanning single-sensor multi-distance measurement approach is shown in Figure 4. As the rotation unit, the rotary table of a Leitz PMM-F 30.20.7 portal coordinate machine is deployed, on which the gear is positioned by a specific clamping unit (cf. Figure 4). The rotation speed of the rotary table amounts to 1.1 min^−1^ and is selected to ensure an almost uniform movement as well as a fast scanning measurement. To measure the contour of the tooth flanks, a confocal-chromatic sensor IFS2405-10 from MicroEpsilon is set up. The sensor has a measuring range of 10 mm at a working distance of 50 mm and is specified with a distance uncertainty of ≤2.5 μm (*k* = 1). The measuring spot has an average diameter of 16 μm on the tooth surfaces. The left tooth flanks are measured. The sensor is positioned so that the entire measuring range is used when detecting the tooth flanks. To align the sensor tangentially to the nominal base circle of the gear, a manual rotary unit is used. Due to a wobble resulting from an eccentric and possibly slightly inclined clamping of the gear on the rotary table, the real sensor alignment deviates from the ideal alignment, cf. Section 2.3.

For the validation of the model-based measuring principle, the sensor system must be calibrated and adjusted. Therefore, the calibration gear is mounted nearly concentric on the clamping unit on the rotary table. A challenge for the set-up is a reproducible clamping of the calibration gear and the gear to be measured afterwards. The similarity condition must also be fulfilled concerning the gear positioning for subsequent measurements. Otherwise, the systematic deviation due to the unknown sensor arrangement will be under- or overcompensated due to the influence of a different wobble. With a suitable gear clamping unit, the influence of the wobble can be significantly reduced. When the measuring system is calibrated (cf. Section 3.3), the gear to be measured can be positioned on the rotary table. Thus, the design of the measurement object and sensor arrangement is optimized to reduce systematic influences as much as possible. Note that all measurements are performed in an air-conditioned measuring room. Temperature changes over time are less than 0.4 K per hour. Besides, the measuring arrangement is set up hours before the measurements to obtain a temperature distribution that is as stationary as possible during the measurements. Therefore, a thermal drift of the used sensor and material expansion of the gear are negligible.

### 3.3. Data Acquisiton and Signal Processing

The profile contour of a gear is detected by using the model-based scanning multi-distance approach. The single confocal-chromatic distance sensor measures the distance to the gear surface with a measuring rate of 1 kHz while the gear is rotated by the rotary table with a rotation speed of 1.1 min^-1^. The distance is determined by integrating the intensity of the reflected wavelengths over the light spot of the confocal-chromatic sensor (diameter = 16 μm). Depending on the surface curvature and sensor alignment to the surface, a linearity error in the distance measurement can occur. However, the linearity error is included in the specified distance uncertainty of 2.5 μm and is therefore considered. Repeated measurements are always started from the same tooth on the left flank side. Per tooth, 1350 measuring points are detected according to the optical accessibility. Due to the shading caused by adjacent teeth, only half of the involute is detected at the outer end up to the tooth tip. Acceleration and braking processes must be taken into account when rotating the rotary table. For the measurement of the tooth flank contours, a pre- and post-run during rotation must be carried out so that the rotational speed remains as constant as possible during the measurement.

**Figure 5:**
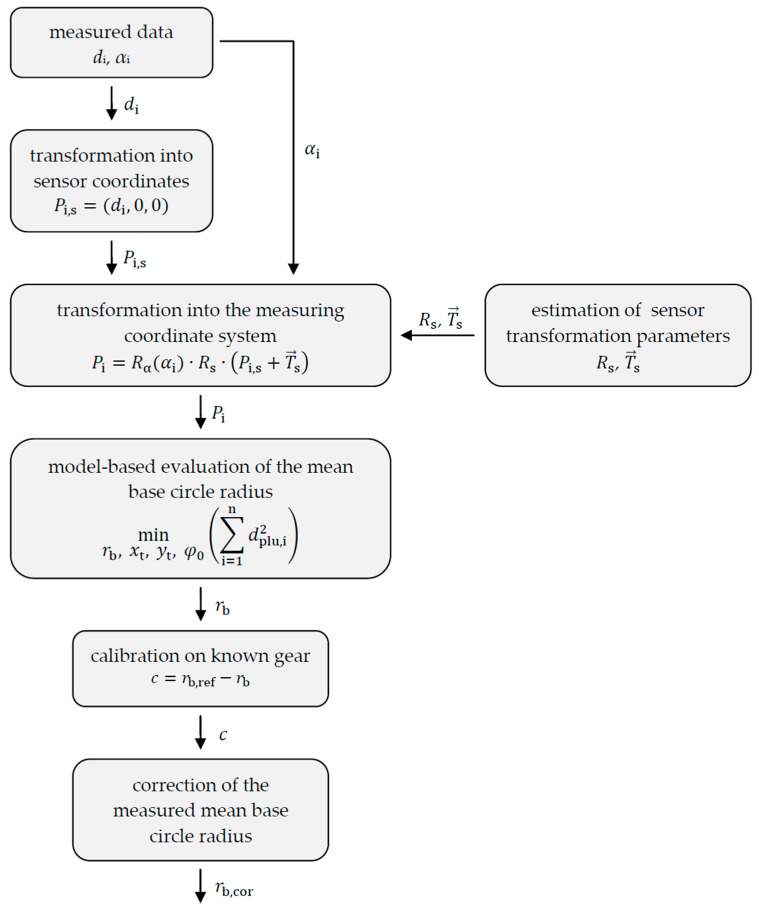
Signal processing to determine the actual mean base circle radius of a gear by calibrating the sensor system with a known gear.

The signal processing of the measured data to obtain the mean base circle radius is shown in Figure 5. For the model-based evaluation of the shape parameter mean base circle radius, the angle-dependent distances $d_{i}$ must be transformed into coordinates. In the first step, sensor coordinates *P*_i,s_ are calculated. Further, the sensor coordinates are transformed into measuring coordinates *P*_i_ according to the transformation of the sensor arrangement to the rotary table ($R_{s}$, ${\vec{T}}_{s}$) and the rotation matrix *R*_α_ of the rotation angles *α*_i_. Since the exact sensor arrangement is not known, the transformation parameters ($R_{s}$, ${\vec{T}}_{s}$) will be estimated. Transformations with deviating estimated sensor positions and alignments lead to a systematic error when determining the mean base circle radius. For this reason, the model-based scanning multi-distance measurement approach is calibrated with a known gear to compensate for the resulting systematic error of the mean base circle radius by a constant correction value *c* (offset correction). A respectively corrected mean base circle radius *r*_b,cor_ then results.

## 4. Results

Due to the unknown sensor arrangement and the resulting systematic error, the model-based scanning multi-distance measurement approach must be calibrated and adjusted first. For this purpose, the geometry of a known calibration gear is measured, compared with the reference geometry using the example of the shape parameter mean base circle radius and a correction value is calculated based on the offset (cf. Section 3.3). For the calibration of the measuring system, a mean correction value *c* = (1.4 ± 0.9) μm (*k* = 1) for the mean base circle radius is calculated from 22 repeated measurements with the same boundary conditions regarding sensor position, alignment and gear position on the rotary table.

The model-based single-sensor multi-distance measurement approach is validated on a second gear with the same nominal geometry. The gear is positioned and clamped on the rotary table according to the calibration measurements. Despite a special clamping device, it cannot be assumed that the gear position will exactly match that of the calibration gear. This must be taken into account when evaluating the shape parameter mean base circle radius. In 22 repeated measurements with constant boundary conditions, the left tooth flank contours are acquired analogous to the calibration measurements. An actual mean base circle radius is then calculated and corrected with the mean correction value *c* from the calibration measurement.

**Figure 6:**
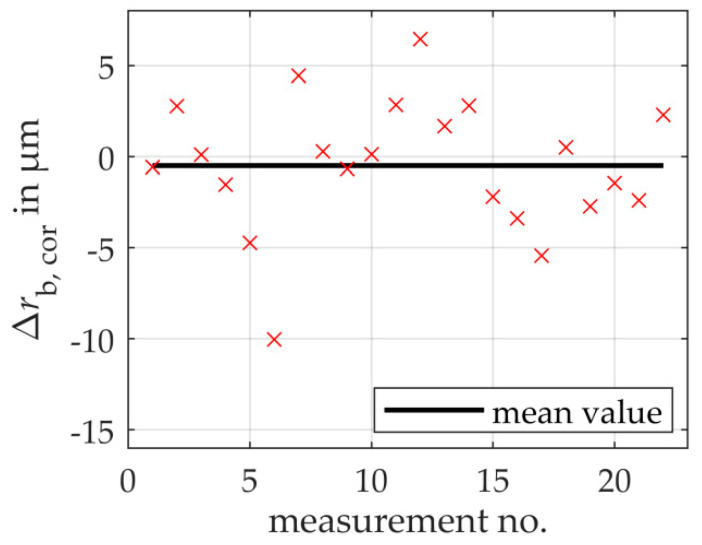
Experimental results of the remaining deviations of the determined mean base circle radii to the corresponding reference base circle radii for repeated measurements on the measured gear. On average, no significant systemic error is found.

Figure 6 shows the measurement deviations of the measured mean base circle radius from the reference base circle radius as a function of the 22 repeated measurements for the measured gear. As a result, taking into account the uncertainty of the reference measurement of 0.5 μm (*k* = 1) as well as the uncertainty of the calibration measurement of 0.9 μm (*k* = 1), the total measurement uncertainty is 3.74 μm (*k* = 1). Thus, the aim of a base circle radius uncertainty < 5 μm is achieved.

The standard measurement uncertainty for the repeated measurements is determined to 3.6 μm. On average, the 22 repeated measurements deviate from the reference value of the second gear by −0.49 μm. This systematic error is assumed to be a superposition of the influences of the changed gear position of the reference gear to the measured gear on the rotary table, the partially different actual geometry and the total measurement uncertainty of the correction value *c*. Concerning the standard measurement uncertainty of the mean value of 0.77 μm of the 22 repeated measurements, the systematic error is not significant. Furthermore, the systematic error is within the standard measurement uncertainty of the reference base circle radius of 0.5 μm. Hence, no significant systematic error exists and the random error dominates the total measurement uncertainty.

An uncertainty contribution to the random error is the distance uncertainty of the confocal-chromatic sensor. The respective contribution to the measurement uncertainty of the mean base circle radius can be estimated using Monte-Carlo simulations. With a distance uncertainty of 2.5 μm, an uncertainty contribution of 0.27 μm (*k* = 1) results for *N* = 100 repetitions. The contribution resulting from the distance uncertainty is thus one order of magnitude smaller than the standard measurement uncertainty of the 22 repeated measurements on the measured gear. A primary contribution to the random error is assumed to be the control of the rotary table. The angle of rotation of the rotary table is not detected synchronously with the corresponding distance information of the confocal-chromatic sensor. A constant angular velocity is assumed over the entire circumference to correlate the measured distances and rotation angles. Due to the control of the rotary table, however, an inconstant angular velocity scattered around a mean value must be assumed. Measured distances are therefore transformed distorted, which leads to local deviations of the measured tooth flank geometry. It therefore expected that the influence due to the control of the rotary table is significantly higher than the influence of the sensor distance uncertainty. In future work, the primarily random error contributions have to be identified and quantified. In summary, the experimental results validate the proof of principle of the model-based scanning multi-distance approach for gear measurements using a confocal-chromatic sensor. Even if the exact contributions to the measurement uncertainty, such as the influence of the rotary table control, are not clarified in detail, uncertainties of < 5 μm are achieved.

## 5. Scalability

To verify the scalability of the model-based scanning multi-distance measuring measurement approach for large gears, the actual mean base circle radius is determined for a simulated non-modified spur gear (cf. Table 1) in 100 repeated measurements using Monte-Carlo simulation, compared with the actual reference geometry, and the corresponding uncertainty is calculated. For each tooth, ≈500 distances to the left tooth flanks are acquired, converted into coordinates and evaluated using the model-based approach. Due to the unknown sensor arrangement and the resulting systematic error in the base circle radius calculation, a correction value *c* must first be determined using a simulated calibration measurement on a known calibration gear (cf. Table 1).

**Figure 7:**
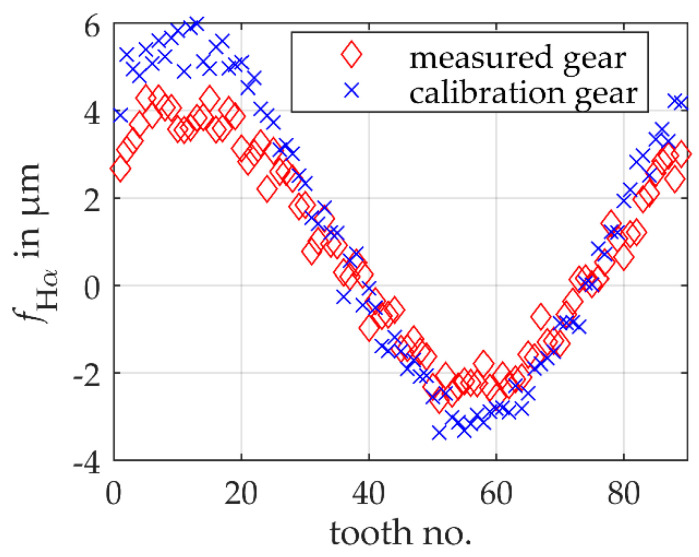
Profile slope deviations $f_{H\alpha }$ of the individual teeth of two simulated non-modified involute spur gears.

To simulate a realistic measurement, the simulated gears are subject to tooth individual profile slope deviations (cf. Figure 7). The sizes of the profile slope deviations are selected according to the permissible tolerances of gear quality grade 5. The profile slope deviations are distributed sinusoidally over the circumference of the gears and superimposed with a normally distributed noise with a standard deviation of 0.35 μm. It should be noted that these are only simulated production-related shape deviations and are therefore not directly comparable with the deviations in Section 3.1. The similarity conditions according to ISO 15530 are met.

A distance sensor with a measuring range of 50 mm and a normally distributed distance uncertainty with a standard deviation of 5 μm are simulated. As in the experiment, the sensor orientation deviates from an ideal tangential alignment on the base circle radius (cf. Section 3.2). The exact positioning of the sensor is also assumed to be unknown for the calculation of the actual mean base circle radius. The exact alignment and positioning of the confocal-chromatic sensor are not known in the experiment. Therefore, the deviations from the ideal sensor arrangement are estimated for the simulations. Furthermore, an ideal rotary table with constant rotational speed is simulated. An influence of the rotary table control is not considered because the real control behavior of the rotary table is not known at present. According to the experimental set-up and concerning a real measurement, an eccentric positioning of the gears on the rotary table is also simulated. Positional deviations of Δx=−10 μm and Δy=50 μm of the simulated gears (calibration gear to measured gear) are chosen according to the experiment to fulfill the similarity conditions.

To calculate the offset correction value *c*, a single measurement with an ideal sensor is simulated. Therefore, uncertainties in the distance measurement are neglected for the calculation of the correction value, to emulate a calibration measurement of multiple repetitions. The offset correction value for the simulated set-up is *c* = 5.4230 mm.

The model-based scanning multi-distance measurement approach for determining the base circle radius as a fundamental gear shape parameter is verified on the simulated gear to be measured. In 100 repeated measurements with constant boundary conditions analogous to the calibration measurement, the left tooth flank contours are acquired. An actual mean base circle radius is then calculated and corrected with the correction value from the simulated calibration measurement. A comparison with the simulated reference geometry (the actual geometry) is considered.

**Figure 8:**
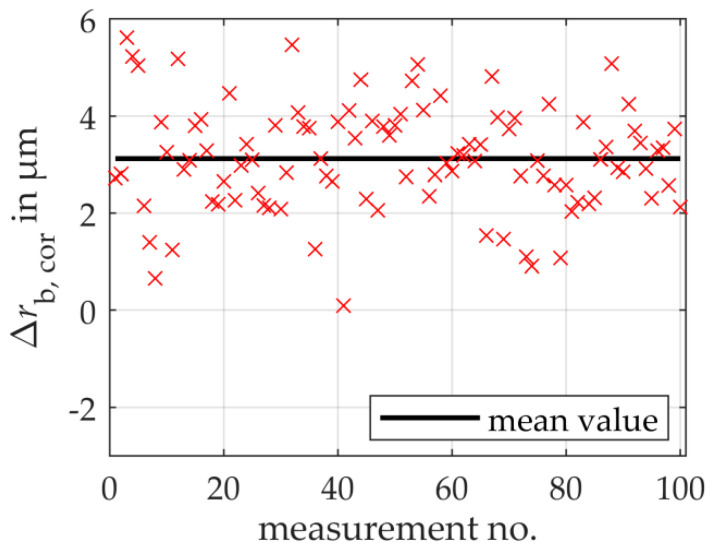
Simulated results of the remaining deviations of the determined mean base circle radii to the corresponding reference base circle radii for repeated measurements on a simulated gear to be measured. On average, a systematic error dominates the total measurement uncertainty.

Figure 8 shows the remaining deviations of the determined mean base circle radius to the reference base circle radius after correction as a function of the 100 repeated simulations on the gear to be measured. As a result, the total measurement uncertainty is estimated to 3.29 μm (*k* = 1), which verifies the model-based scanning multi-distance measurement approach regarding the selected simulation boundary conditions.

The standard measurement uncertainty of the random errors for the 100 repeated simulations amounts to 1.1 μm and results from the distance uncertainty of the simulated sensor. The repeated measurements deviate on average by 3.1 μm from the reference value of the actual base circle radius. The systematic error is three times higher than the random error and thus dominates the total measurement uncertainty compared with the experimental results. Further Monte-Carlo simulations provide information about the measurement uncertainty contributions due to the different actual geometries and position deviation of the simulated gears to the systematic error. In a first simulation, the influence of the different actual geometries is examined under the assumption of an ideal sensor and an ideal positioning of the gear identical to the simulated calibration measurement. The measurement uncertainty contribution amounts to 1.8 μm. Besides, the influence of the position deviation of the simulated gears is characterized. For this purpose, the calibration gear is again considered. Assuming an ideal sensor, the calibration gear is positioned identically to the simulated gear to be measured on the simulated rotary table, the base circle radius is determined and compensated with the previously calculated correction value. The measurement uncertainty contribution to the systematic error is 1.3 μm. Concerning the systematic error of 3.1 μm determined in the repeated simulations (cf. Figure 8), it is clear that the respective influences of shape deviations and gear position deviations correlate with each other. It can be seen that when the gears are scaled to diameters ≥ 1 m, the systematic error is not completely compensated by the calibration.

The aim of achieving a total measurement uncertainty of less than 5 μm seems feasible for large gears. A carefully arranged sensor set-up indicates a systematic error < 5 μm, even if the size of the gear exceeds 1 m. When the gear size is changed, the number of teeth varies. This must be taken into account when determining the total measurement uncertainty. However, the number of teeth influences the random error, provided that mean gear parameters such as the mean base circle radius are calculated. The influence of the number of teeth is not significant compared with the systematic influences. Further systematic and random influences are to be expected in the experiment, for example, from the real measurement arrangement and the rotary table control, since the simulations consider only the pretended input. Besides, the use of an appropriate calibration gear that meets the similarity conditions must be considered. In summary, the simulated results indicate the suitability and scalability of the model-based scanning multi-distance measurement principle for large gear measurements.

## 6. Conclusions and Outlook

In this article, a novel scanning multi-distance measurement approach with a model-based evaluation of the mean base circle radius as a fundamental shape parameter of gears with an uncertainty of less than 5 μm (*k* = 1) is presented. A confocal-chromatic distance sensor is combined with a rotary table to perform scanning gear measurements, so that geometrical data of all teeth are taken into account when evaluating the shape parameter mean base circle radius. A flexible sensor arrangement enables a scalable measuring volume to measure even larger gears in the future.

An unknown sensor arrangement of the model-based scanning multi-distance measurement approach leads to a systematic error in the base circle radius calculation. To compensate for the systematic error, a calibration strategy using a known calibration gear is presented. The measuring principle is validated experimentally on a midsize gear by comparison with reference measurements in a preliminary study.

As a result, the total measurement uncertainty of the mean base circle radius is <5 μm. The experimental results thus validate the model-based scanning multi-distance measurement approach for gear measurements. No significant systematic error is detected in the repeated measurements, so that the random error dominates the total measurement uncertainty. One reason for the random error could be the assumption of a constant angular velocity of the rotary table, which was made due to the unknown control behavior of the rotary table and leads to distortions in the transformation of the distances into coordinates. In particular, the experimental results demonstrate the suitability of the confocal-chromatic sensor for gear measurements. Despite complex measurement requirements due to the curved and bright surface of the gear involutes, the mean base circle radius of midsize spur gears can be determined with an uncertainty of < 5 μm using the confocal-chromatic sensor.

Simulations show that a total measurement uncertainty for the mean base circle radius of <5 μm can be achieved for a gear with a diameter of ≥1 m. Hence, the simulation results verify the scalability of the model-based scanning multi-distance measurement approach for large gear measurements regarding the selected boundary conditions. In comparison with the experiments, the estimated total measurement uncertainty is dominated by a systematic error. Further developments are in progress to improve the model-based scanning multi-distance measurement approach and to reduce the total measurement uncertainty.

The experimental and simulation results of the preliminary study also prove the applicability of the presented calibration strategy. For small to midsize gears, no significant systematic error remains after calibration, whereas for large gears, a dominating systematic error remains concerning the total measurement uncertainty.

In future work, large gear measurements will be performed to validate the scalability of the model-based scanning multi-distance measurement approach, since the measurement conditions concerning the sensor arrangement, handling and positioning of the gear on the rotary table are different from those for small to midsize gears. The suitability of the confocal-chromatic sensor for large gear measurements will also have to be tested in further measurements, since only a small part of the involute is measured due to the limited measuring range. The influences on the base circle radius calculation have to be characterized. Moreover, the calibration strategy for large gear measurements is to be extended, since the current remaining systematic error in large gear measurements contributes a considerable component to the total measurement uncertainty. Further, the measurability of further gear shape parameters with the model-based scanning multi-distance measurement approach must be investigated in the future to achieve extensive gear quality assessments. The demonstrated geometric model allows, for example, an extension of the determination of the profile slope deviation. For the implementation, an adjustment of the evaluation algorithm is necessary, which is subject of ongoing research. The first results indicate the feasibility to determine the profile slope deviation.

## Figures and Tables

**Table 1:** Geometric parameters of the simulated gears.

	Number of Teeth	Normal Module in mm	Nominal Base Circle Radius in mm	Actual Base Circle Radius in mm
measured gear	89	12	501.7959	501.8035
calibration gear	89	12	501.7959	501.8011
